# Systemic Inflammation in Retinal Vein Occlusion and Its Role in Clinical Outcomes of Secondary Macular Edema

**DOI:** 10.3390/jcm15020407

**Published:** 2026-01-06

**Authors:** Elsa Wilma Böhm, Peter Wolfrum, Anna Maria Welzel, Alexander K. Schuster, Felix M. Wagner, Adrian Gericke, Katrin Lorenz, Bernhard Stoffelns, Christina A. Korb

**Affiliations:** Department of Ophthalmology, University Medical Center Mainz, Johannes Gutenberg University Mainz, Langenbeckstrasse 1, 55131 Mainz, Germany

**Keywords:** retinal vein occlusion, macular edema, inflammation, C-reactive protein, homocysteine

## Abstract

**Objectives**: This study aims to analyze the association between systemic inflammation and clinical outcomes in patients with macular edema secondary to retinal vein occlusion (RVO). **Methods**: A retrospective analysis of 68 patients with acute RVO, in whom cardiovascular risk factors were assessed, was conducted. Laboratory determinations included levels of C-reactive protein (CRP) and homocysteine to explore the systemic inflammatory status. Optical coherence tomography (OCT) parameters and visual acuity (LogMAR) were collected at baseline. Follow-up visual acuity was determined after 12 months. The number of intravitreal anti-VEGF injections and the performed laser treatment were assessed after 12 and 24 months. Associations of inflammatory markers with clinical outcomes were analyzed by correlation analysis, and patients with or without evidence for inflammation were compared. **Results**: At baseline, the mean foveal retinal thickness (FRT) was 591 ± 277 µm, the mean average central retinal thickness (CRT) was 580 ± 227 µm, and the mean average central retinal volume (CRV) was 12 ± 3 mm^3^. The level of CRP at baseline was significantly associated with increased FRT, average CRT, and average CRV (*p* = 0.024; *p* = 0.027; *p* = 0.003). CRP levels were also associated with a lower BCVA at baseline and after 12 months (*p* = 0.018; *p* = 0.006). In patients with elevated homocysteine levels, a trend towards a higher number of required laser treatments was observed. No association between increased inflammatory parameters and the number of required intravitreal injections was detected. **Conclusions**: Systemic inflammation is associated with the severity of macular edema secondary to RVO. CRP concentrations could represent a prognostic marker for the course of the patient’s visual acuity.

## 1. Introduction

Retinal vein occlusion (RVO) is the second most prevalent retinal vascular disease, affecting about 28.06 million people worldwide [1]. A prevalence of about 0.4% was estimated in the German population [2]. By obstruction of either the central, hemi-central, or branch retinal vein as a consequence of impaired retinal blood flow, compromised retinal vascular endothelium, and alteration of blood composition, venous retinal blood flow is interrupted with a subsequent increase in capillary pressure. This causes increased vascular permeability, leading to vascular leakage of blood cells and fluid into the retina. Thereby, macular edema occurs, which is the main cause of visual impairment secondary to RVO. The subsequent release of growth factors, such as vascular endothelial growth factor (VEGF)-A, as a response to retinal ischemia during RVO aggravates vascular damage and functional deterioration [3]. Intravitreal injections of anti-VEGF agents with regular monitoring of visual acuity and morphologic parameters on optical coherence tomography (OCT) represent the standard therapy to date [3]. In cases of ischemic conversion with non-perfusion areas or pathologic neovascularization, retinal laser photocoagulation aiming at reducing retinal oxygen demand is indicated, thereby diminishing the stimulus for pathologic neovascularization due to VEGF release [3].

Although the association of RVO with cardiovascular risk factors, such as arterial hypertension and dyslipidemia, has been reported by several studies, the individual cause of RVO often remains unknown, and reliable prognostic markers are lacking [2,4].

The identification of novel risk factors and prognostic markers is important to improve therapeutic regimens and patient outcomes. Systemic inflammation is, on the one hand, a pathogenic driver of increased thrombotic risk and, on the other hand, involved in the promotion of vascular permeability, both being key players in the pathogenesis of RVO and related macular edema. Thus, studies on the role of systemic inflammation during RVO represent an emerging scientific field [5,6,7,8,9].

Pro-inflammatory markers, such as interleukin-6 (IL-6) or monocyte chemotactic protein 1 (MCP-1), were found to be increased in the vitreous fluid of patients with RVO. Additionally, these levels of inflammatory factors correlated with retinal vascular permeability and the severity of macular edema in patients with RVO, indicative of a crucial role of inflammation in the pathogenesis of macular edema [10,11]. However, since the collection of samples of vitreous fluid is invasive and can be related to complications, such as intraocular infections or bleeding, the establishment of more less-invasive examinations, such as blood sampling, is required to explore the role of inflammation in patients with RVO. CRP is a standardized measurable laboratory parameter for systemic inflammation, belonging to clinical routine for the monitoring of infectious or rheumatic diseases [12]. It belongs to the family of acute-phase reactant proteins, synthesized by the liver and released during acute and chronic inflammation after stimulation through IL-6 secreted by macrophages and T-cells [12,13]. In addition to its role in the monitoring of infections, its association with cardiovascular diseases and its potential to predict cardiovascular events is increasingly gaining attention [14,15,16]. Thereby, the direct contribution of systemic inflammation to vascular damage during several vascular diseases is probable.

Homocysteine is a non-proteinogenic amino acid with a sulfhydryl group that is an intermediate of methionine metabolism. Due to the activation of inflammatory pathways, oxidative stress, and endothelial damage, it aggravates atherosclerosis and cardiovascular risk [17]. Hyperhomocysteinemia has been identified as a potential risk factor for RVO, but less is known about its impact on clinical outcomes and its potential as a prognostic marker in RVO [18,19].

This analysis aims to explore the impact of systemic inflammation assessed by serum levels of CRP and homocysteine in RVO and their relation to morphological parameters and functional and therapeutic outcomes.

## 2. Materials and Methods

### 2.1. Patient Selection

A post hoc analysis of patients who were admitted to the Department of Ophthalmology of the University Medical Center Mainz, Germany, for inpatient assessment of risk factors after RVO was conducted. Patients with macular edema secondary to RVO who were treated as inpatients between May 2014 and August 2024 were considered for inclusion in the analysis (68 patients). Only patients with symptom onset within two weeks were included in the study. During their hospital stay, all patients underwent blood sampling on the first day of their hospital stay, ophthalmic examination, and an assessment of cardiovascular risk factors, and they received the first intravitreal injection of anti-VEGF agents in the case of indicated treatment for macular edema secondary to RVO, also on the first day of their inpatient stay. After discharge, patients were treated in 4–6 weekly intervals with intravitreal anti-VEGF injections according to the pro-re nata scheme of clinical routine. Patients with missing relevant data, continuation of intravitreal anti-VEGF treatment after discharge to another ophthalmologic center, or presence of other retinal comorbidities, such as diabetic retinopathy or myopic choroidal neovascularization, were excluded from analysis.

As this was a retrospective analysis of clinical care data, no ethical approval or patient consent was required. For publication, only anonymized data were obtained, and no third parties were able to obtain insights into the original health data of individual patients. This type of investigation complies with legal requirements (“Landeskrankenhausgesetz” §§36–37).

### 2.2. Data Collection

Data were independently evaluated by two ophthalmologists (E.W.B. and C.A.K.). The baseline characteristics were collected during the inpatient stay and included age, sex, laterality of RVO, type of RVO (central RVO, hemi-central RVO, or branch RVO), lens status (phakia or pseudophakia), ocular comorbidities, such as glaucoma, and systemic comorbidities, such as arterial hypertension.

Decimal best-corrected visual acuity was collected at baseline and after 12 months and converted to a LogMAR scale, as reported by Beck et al. [20].

For morphological parameters, spectral domain-OCT (SD-OCT) volume scans (Spectralis^TM^, Heidelberg Engineering, Heidelberg, Germany) were analyzed at baseline on the first day of their inpatient stay. Foveal retinal thickness (FRT) was manually measured using the measurement tools of the Heyex 2 software (Heidelberg Engineering, Heidelberg, Germany). Additionally, the average central retinal thickness (CRT) in the central 1 mm^2^ area of the macula and the average central retinal volume (CRV) in the central 6 mm^3^ area of the macula were assessed. For comparability, the automatic tracking function of Heyex 2 was used. Laboratory results for CRP and homocysteine were assessed at baseline during the inpatient stay. Blood sampling was performed in the morning of the first day of their inpatient stay, and it is routinely performed in the fasting state at our institution. Levels of CRP (mg/L) and homocysteine (µmol/L) were measured according to clinical routine by the Institute of Clinical Chemistry and Laboratory Medicine of the University Medical Center Mainz. C-reactive protein levels were measured with the Alinity c analyzer (Abbott Diagnostics, Abbott Park, IL, USA), performing an immunoturbidimetric assay (CRP Vario Reagent Kit). Quality control was conducted with Liquicheck Immunology Control levels 1–3 (Bio-Rad, Hercules, CA, USA). The lower limit of quantification was 0.2 mg/L. CRP levels ≥ 5 mg/L were considered abnormal. Homocysteine was measured with the Alinity i analyzer (Abbott Diagnostics, Abbott Park, IL, USA) using a chemiluminescent microparticle immunoassay (CMIA; Alinity i Homocysteine, Abbott, Abbott Park, IL, USA). Internal controls provided by Abbott were used. The lower limit of quantification was 1 µmol/L. Homocysteine levels ≥ 15 µmol/L were considered abnormal.

### 2.3. Intravitreal Injection Procedure

All patients included in the study received intravitreal anti-VEGF injections according to a pro-re nata scheme of clinical routine. Patients with macular edema secondary to RVO received an initial triple series in 4–6 weekly intervals with the first injection within two weeks after onset of symptoms. In the case of persistent or recurrent clinically significant macular edema, further injections were administered.

After anesthesia of the ocular surface with 1% tetracaine eye drops, the surrounding skin was disinfected with 10% povidone-iodine. The eyelid was kept open by an inserted eyelid speculum, and the ocular surface, including conjunctiva and cornea, was disinfected with 5% povidone-iodine. The anti-VEGF agents (bevacizumab, aflibercept, or ranibizumab) were applied into the vitreous cavity using a 30-gauge needle through the pars plana. During the study period, different anti-VEGF agents were used according to clinical practice. In the case of insufficient therapeutic response in terms of visual acuity, macular edema morphology, or intolerance to one anti-VEGF agent, a switch to another agent was performed.

### 2.4. Retinal Photocoagulation

All patients received fluorescein angiography at baseline and during follow-up examinations every 6 months. In the case of anterior segment neovascularization, neovascularization of the disc, or retinal neovascularization elsewhere, pan-retinal photocoagulation was performed. Clinically relevant retinal non-perfusion areas were also considered as ischemic conversion and treated with retinal photocoagulation.

### 2.5. Statistical Analysis

Statistical analysis was performed by using the statistical software GraphPad Prism 10.

For the association between levels of CRP or homocysteine and morphological parameters and visual acuity, Pearson or Spearman correlation tests were conducted. Furthermore, multivariable regression analysis was performed. To compare the levels of systemic inflammatory parameters in different RVO types, Tukey’s multiple comparison test was used. Mean values were compared using an unpaired *t*-test for parametric data, and the Mann–Whitney U test was used for non-parametric data. The level of significance was set at *p* < 0.05.

## 3. Results

### 3.1. Descriptive Data

A total of 68 eyes of 68 patients with macular edema secondary to RVO were included, of whom 33 were female (48%) and 35 were male (52%). The mean age was 64.5 ± 12.1 years. The right eye was affected in 28 patients (41%), and the left eye was affected in 40 patients (59%). Central RVO occurred in 33 patients (48%), hemi-central RVO occurred in 10 patients (15%), and branch RVO occurred in 25 patients (37%). The severity of macular edema was assessed by morphological parameters on OCT at baseline. The mean FRT measured 591 ± 277 µm, the average CRT measured 580 ± 227 µm, and the average CRV measured 12 ± 3 mm^3^. The mean CRP levels amounted to 3.70 ± 4.58 mg/L, while CRP levels ≥ 5 mg/L are considered abnormal. The mean homocysteine levels were 11.49 ± 4.70 µmol/L. Hyperhomocysteinemia was defined by homocysteine levels of ≥15 µmol/L.

Visual acuity in LogMAR at baseline was 0.73 ± 0.61 and improved to 0.46 ± 0.40 after 12 months. Patients received a total of 7.5 ± 2.4 intravitreal injections within the first 12 months of treatment and 13.7 ± 4.7 within the first 24 months of treatment.

The descriptive data are summarized in Table 1.

### 3.2. Inflammatory Parameters in Different Types of Retinal Vein Occlusion

**Figure 1:**
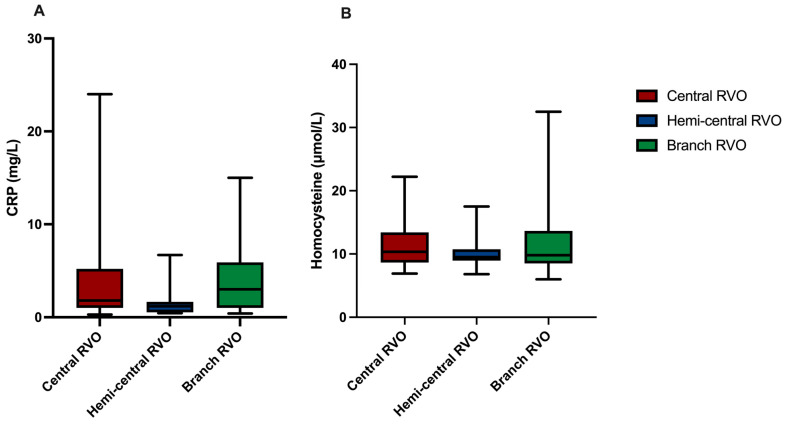
Levels of C-reactive protein and homocysteine in different types of retinal vein occlusion. Levels of C-reactive protein (CRP, in mg/L) showed no differences between central retinal vein occlusion (RVO), hemi-central RVO, and branch RVO (**A**). Accordingly, levels of homocysteine (in µmol/L) did not differ between central RVO, hemi-central RVO, and branch RVO (**B**) (Tukey’s multiple comparison test, *p* > 0.05).

The mean CRP measured 4.09 ± 5.49 mg/L in patients with central RVO, 1.63 ± 1.85 mg/L in patients with hemi-central RVO, and 4.10 ± 3.89 mg/L in those affected by branch RVO. There was no statistically significant difference in CRP levels between different types of RVO (central RVO vs. hemi-central RVO: *p* = 0.306; central RVO vs. branch RVO: *p* = 0.999; hemi-central RVO vs. branch RVO: *p* = 0.331). These data are visualized by box plots in Figure 1A.

Twenty-seven % of all patients had CRP levels of ≥5 mg/L, and 26% of those had central RVO, 10% of those had hemi-central RVO, and 35% of those had branch RVO. These findings indicate that there is no association between CRP levels and the size of venous occlusion.

The mean homocysteine level amounted to 11.31 ± 3.81 µmol/L in patients with central RVO, 10.23 ± 3.00 µmol/L in those with hemi-central RVO, and 12.24 ± 6.08 µmol/L in patients with branch RVO. We could not find any difference concerning homocysteine levels between the three types of RVO (central RVO vs. hemi-central RVO: *p* = 0.826; central RVO vs. branch RVO: *p* = 0.781; hemi-central RVO vs. branch RVO: *p* = 0.539; Figure 1B).

In total, 16% of all patients had homocysteine levels of ≥15 mol/L. When differing between the different RVO types, 11% in central RVO, 11% in hemi-central RVO, and 19% in branch RVO were considered to have hyperhomocysteinemia.

### 3.3. Impact of Inflammatory Markers on Morphological Parameters

**Figure 2:**
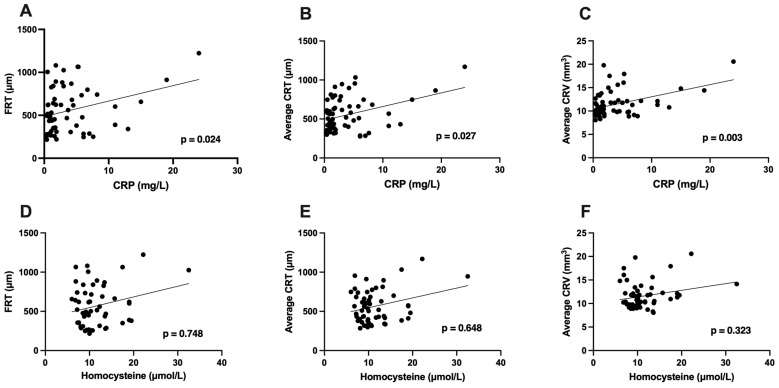
Correlation analysis of C-reactive protein and homocysteine levels with morphological parameters on optical coherence tomography. Correlation analysis revealed a positive association between levels of C-reactive protein (CRP, in mg/L) and foveal retinal thickness (FRT, in µm) (**A**), average central retinal thickness (CRT, in µm) (**B**), and average central retinal volume (CRV, in mm^3^) (**C**). There was no association between homocysteine levels (in µmol/L) and FRT (**D**), average CRT (**E**), and average CRV (**F**).

Correlation tests revealed a significant association between CRP levels and the severity of macular edema, assessed by FRT (*p* = 0.024), average CRT (*p* = 0.027), and average CRV (*p* = 0.003) on OCT (Figure 2A–C). After adjusting for patients’ age, sex, and type of RVO, similar results were found for CRT (*p* = 0.015) and CRV (*p* < 0.001) but not for FRT (*p* = 0.056). The mean FRT measured 655 ± 316 µm in patients with CRP levels ≥ 5 mg/L and 521 ± 236 µm in those with CRP levels < 5 mg/L, without a statistically significant difference between the two groups (*p* = 0.149). Analyses of mean average CRT with 644 ± 279 µm in patients with CRP ≥ 5 mg/L and 520 ± 179 µm in those with normal CRP levels also revealed no difference between the two groups (*p* = 0.169). Remarkably, the average CRV was significantly higher in patients with elevated CRP levels (13 ± 3 mm^3^) compared to those with normal CRP levels (11 ± 2 mm^3^) (*p* = 0.045). When differentiating between different RVO types, in central RVO, correlation analysis revealed comparable results for FRT (*p* = 0.017) and CRT (*p* = 0.007), while no association between CRP and morphological parameters was shown in patients with hemi-central RVO. In branch RVO, a significant correlation of CRP levels with average CRV was found (*p* = 0.004).

Homocysteine levels were not significantly associated with average FRT, CRT, and CRV (*p* = 0.748; *p* = 0.648; *p* = 0.323) (Figure 2D–F). The same results were found after adjusting for patients’ age, sex, and type of RVO (*p* = 0.349; *p* = 0.304; *p* = 0.694). In patients with hyperhomocysteinemia, the mean FRT (702 ± 326 µm) and average CRT (697 ± 286 µm) did not differ significantly from those with homocysteine levels of <15 mL/L (543 ± 239 µm; 540 ± 185 µm) (*p* = 0.190; *p* = 0.125). In the grouped comparison of patients with hyperhomocysteinemia and patients with normal homocysteine levels, the average CRV was significantly increased in patients with hyperhomocysteinemia (14 ± 3 mm^3^) in comparison to those with normal homocysteine levels (11 ± 3 mm^3^) (*p* = 0.010). In a separate analysis of different RVO types, we found a significant association between homocysteine levels and average CRT in those with central RVO (*p* = 0.045) and branch RVO (*p* = 0.040). There was no significant association between homocysteine levels and morphological parameters in patients with hemi-central RVO.

### 3.4. Impact of Inflammatory Parameters on Visual Acuity

Correlation tests revealed a positive association between baseline CRP levels and visual acuity in LogMAR at baseline (*p* = 0.018) and after 12 months of follow-up (*p* = 0.006), indicative of an adverse association of CRP levels on functional outcomes of macular edema secondary to RVO. The same results were found after adjusting for patients’ age, sex, and type of RVO at baseline (*p* = 0.042). After 12 months, data were adjusted for patients’ age, sex, type of RVO, and visual acuity at baseline, and a significant association between CRP and visual acuity after 12 months was found (*p* = 0.010). In a separate analysis of different RVO types, a significant correlation between CRP levels and visual acuity was only found in patients with central RVO at baseline (*p* = 0.020).

In contrast, homocysteine levels showed no association with visual acuity, neither at baseline nor after 12 months of follow-up (*p* = 0.667; *p* = 0.933).

**Figure 3:**
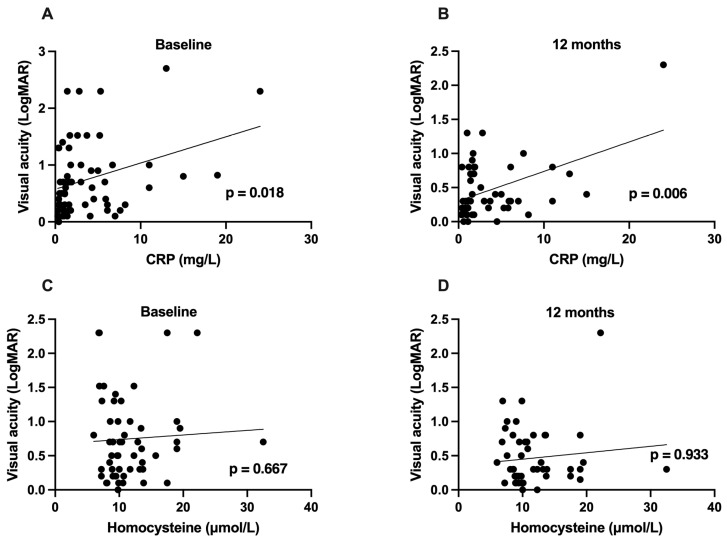
Correlation analysis of C-reactive protein and homocysteine levels with visual acuity. Correlation analysis revealed a positive association between levels of C-reactive protein (CRP, in mg/L) and visual acuity (LogMAR) at baseline (**A**) and after 12 months (**B**). There was no significant association between homocysteine levels and visual acuity at baseline (**C**) and after 12 months of follow-up (**D**).

The data are demonstrated as a scatter plot in Figure 3.

### 3.5. Impact of Inflammatory Parameters on Therapeutic Outcomes

**Figure 4:**
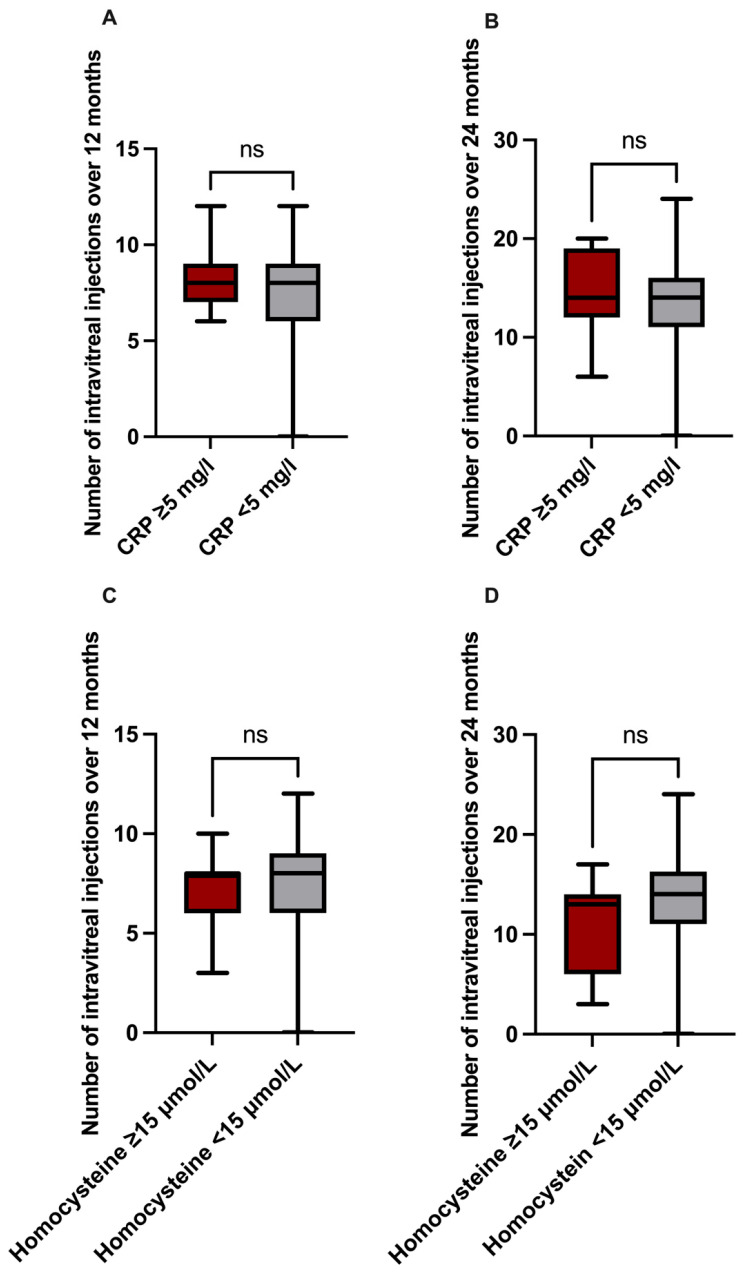
Number of intravitreal injections according to the pro-re-nata scheme in patients with increased and normal inflammatory markers. The required number of intravitreal injections after 12 (**A**) and 24 months (**B**) did not differ between patients with C-reactive protein (CRP) levels ≥ 5 mg/L and <5 mg/L. Additionally, there was no difference concerning the required number of intravitreal injections between patients with hyperhomocysteinemia (homocysteine levels ≥ 15 µmol/L) and those with homocysteine levels < 15 µmol/L after 12 (**C**) and 24 months (**D**) of follow-up (Mann–Whitney U test for non-parametric data or unpaired *t*-test for parametric data, *p* > 0.05).

The mean number of intravitreal anti-VEGF injections after 12 months (8.2 ± 1.8) and 24 months (14.6 ± 4.9) in patients with CRP levels of ≥5 mg/L did not differ significantly from patients exhibiting normal CRP levels (7.4 ± 2.5; 13.4 ± 5.2) (*p* = 0.506; *p* = 0.681; Figure 4A,B).

In accordance with these results, patients with hyperhomocysteinemia also did not require higher numbers of intravitreal anti-VEGF injections, either within 12 (7.1 ± 2.2 versus 7.4 ± 2.5) or within 24 months (11.3 ± 5.0 versus 13.6 ± 5.1), in comparison to patients with normal homocysteine levels (*p* = 0.789; *p* = 0.281; Figure 4C,D).

Moreover, the number of patients requiring retinal photocoagulation did not differ between those with CRP levels of ≥5 mg/L and those with CRP levels of <5 mg/L after 12 (38% versus 33%) and 24 months of follow-up (58% versus 49%).

**Figure 5:**
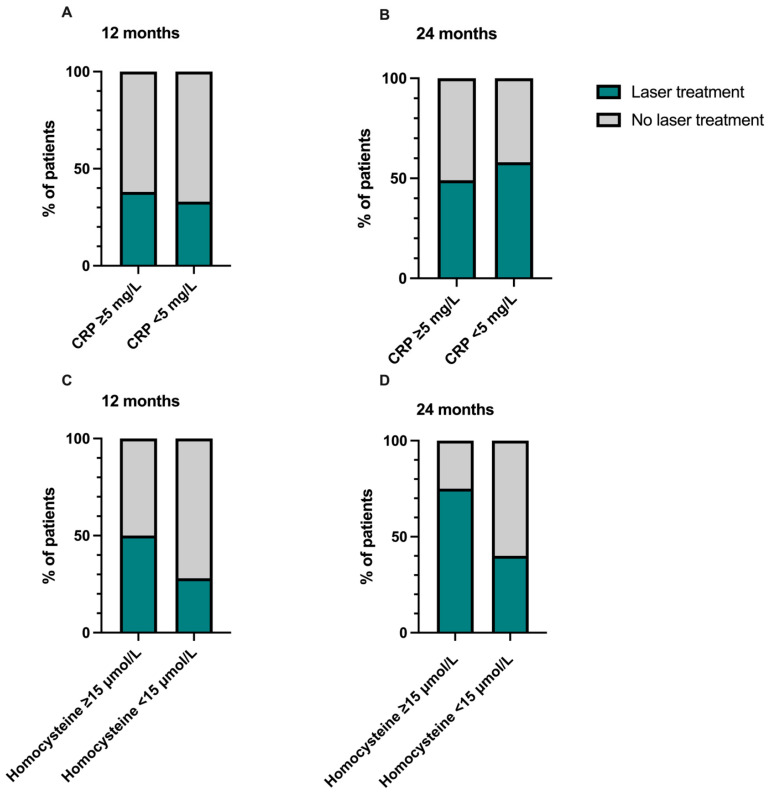
Percentage of patients with required retinal laser treatment. There was no difference in the number of patients that received laser treatment between those with C-reactive protein (CRP) levels ≥ 5 mg/L and those with CRP levels < 5 mg/L after 12 (**A**) and 24 months (**B**). Although a higher percentage of patients requiring laser treatment was observed in those with hyperhomocysteinemia, there was no difference in the number of patients that received laser treatment between those with homocysteine levels ≥ 15 µmol/L and those with homocysteine levels < 15 µmol/L after 12 (**C**) and 24 months (**D**) (Fisher’s exact test, *p* > 0.05). The data is visualized as a percentage (%) of all patients with increased or normal inflammatory markers.

In total, 50% of patients with hyperhomocysteinemia and 28% of patients with regular homocysteine levels received retinal photocoagulation due to ischemic conversion after 12 months. Interestingly, after 24 months of follow-up, laser treatment was required in 75% of patients with hyperhomocysteinemia, while only 40% of those with normal homocysteine levels received laser treatment, indicative of a higher rate of ischemic conversion in patients with hyperhomocysteinemia. However, these findings were without statistical significance (*p* = 0.240; *p* = 0.118), and the small sample size of nine patients with hyperhomocysteinemia might underpower this analysis. This data is visualized in Figure 5.

## 4. Discussion

This study reports the association between systemic inflammation and macular edema secondary to RVO. There are several relevant findings emerging from this single-center tertiary-care setting study. We observed that systemic inflammation, especially assessed by serum levels of CRP, is associated with the increased severity of the height and volume of macular edema secondary to RVO, while systemic inflammatory parameters did not have an effect on the type of venous occlusion. Interestingly, higher CRP levels were associated with worse visual acuity at baseline and after 12 months of follow-up, while homocysteine levels showed no correlation with visual acuity. We also found that these inflammatory parameters did not influence the number of required intravitreal injections of anti-VEGF agents over 12 and 24 months. Notably, patients with hyperhomocysteinemia might require more laser treatments due to retinal non-perfusion and proliferation after 12 and 24 months of follow-up compared to those with normal homocysteine levels, suggestive of a more pronounced ischemic disease phenotype in these patients.

CRP is a well-established marker for systemic inflammation, and its relevance for vascular diseases, including atherosclerosis and thrombosis, both features of RVO, has been demonstrated in different vascular beds [21,22,23,24]. For example, in patients undergoing percutaneous coronary intervention because of myocardial infarction, pre-interventional CRP levels were associated with a higher risk for the development of in-stent restenosis [24]. Interestingly, the CRP levels of patients with multivessel-coronary artery disease were significantly higher than those of patients with single- or double-vessel disease. In addition to CRP levels, increased intima-media thickness of the carotid artery correlated with the severity of coronary artery disease, making both parameters relevant predictors in the severity of atherosclerotic disease [25]. In patients with symptomatic carotid stenosis, high-sensitivity CRP levels were significantly higher than in asymptomatic patients, suggestive of the potential of this marker in identifying patients with an elevated risk of developing neurological deficits [26].

Hyperhomocysteinemia is also associated with inflammation and atherosclerosis. Thereby, it is recognized as an independent cardiovascular risk indicator in various vascular diseases [27]. For example, a meta-analysis revealed that hyperhomocysteinemia is associated with increased risk for stroke [28]. Furthermore, it was identified as a diagnostic marker for coronary artery disease and heart failure [29]. Apart from cardiovascular diseases, hyperhomocysteinemia is also associated with the occurrence of RVO [18,30,31]. Furthermore, a higher risk for RVO was suggested in patients with increased CRP levels [32].

RVO occurs due to the sudden interruption of blood flow in retinal veins by thrombus formation. Since RVO is associated with cardiovascular risk factors such as arterial hypertension and dyslipidemia, it is also considered an atherosclerotic disease [2,4]. Due to the compression of retinal veins by crossing atherosclerotic retinal arterioles, subsequent venous stasis and thrombus formation cause RVO [33]. Subsequently, increased pressure in retinal capillaries and increased capillary permeability lead to the leakage of fluid into the macula. Thereby, macular edema develops, leading to visual impairment. Capillary damage and related macular edema are exacerbated by retinal ischemia that triggers the release of growth factors, such as VEGF-A, a key factor in the pathogenesis of macular edema [3]. In addition to VEGF-A, other studies found several other signaling molecules and pathways involving oxidative stress and inflammation that contribute to the pathogenesis of macular edema in RVO [34,35,36]. Since elevated levels of interleukins, such as IL-6 and IL-8, were detected in the vitreous fluid of patients with RVO, a contribution of inflammation in its pathogenesis is probable [37,38]. Additionally, vitreous fluid levels of VEGF-A and IL-6 correlated with the severity of macular edema, indicative of a crucial role of inflammation in capillary damage and concomitant macular edema [37]. The downregulation of endothelial tight junctions triggered by IL-8, as demonstrated by cell culture studies, might be a major cause of increased capillary permeability in RVO [39]. Additionally, subsequent upregulation of adhesion molecules for immune cells, such as intercellular adhesion molecule 1 (ICAM-1), further aggravates leakage from retinal microvessels and thereby macular edema [36]. A correlation between vitreous fluid inflammatory factors, including VEGF-A, IL-6, ICAM-1, and the acute-phase protein pentraxin (PTX)3, with retinal vascular permeability and the severity of macular edema was also reported by Noma et al. [10]. Interestingly, increased inflammatory and oxidative stress parameters were not only elevated in the vitreous fluid of patients with RVO, but also in the serum, indicative of a systemic role of inflammation in RVO [40]. Furthermore, blood-derived inflammatory parameters, such as the neutrophil-to-lymphocyte ratio, platelet-lymphocyte ratio, and systemic inflammation index, were significantly higher in patients with macular edema secondary to RVO compared to age- and sex-matched healthy controls [41]. These findings were confirmed by other authors who also found higher neutrophil-to-lymphocyte ratios in RVO patients compared to controls [42,43,44]. Blood-count-derived inflammation indices, such as the neutrophil-to-lymphocyte ratio, were even identified as potential predictors for neovascular glaucoma secondary to RVO [45]. There are other studies evaluating the impact of systemic inflammatory parameters on macular edema secondary to RVO. For example, serous retinal detachment in macular edema secondary to RVO, an OCT biomarker for inflammation, could be predicted by the neutrophil-to-lymphocyte ratio and systemic inflammation index. Other authors reported better visual outcomes after intravitreal anti-VEGF therapy predicted by the platelet-to-lymphocyte ratio and neutrophil-to-lymphocyte ratio [46].

Increased CRP levels were associated with the increased severity of macular edema in RVO patients. In patients with central RVO, CRP levels were significantly associated with FRT and average CRT, while in branch RVO, CRP levels were only significantly associated with average CRV. This observation could be explained by the fact that, in central RVO, macular edema is the highest in the center of the macula, while macular edema can be more decentralized depending on the site of venous occlusion. Since we analyzed the retinal volume of the central 6 mm^3^, this parameter might be more valuable for macular edema in branch RVO. CRP levels were also associated with worse visual acuity. The fact that CRP levels at diagnosis were not only associated with worse visual acuity at baseline but also after 12 months indicates that the initial severity of macular edema reduces the long-term prognosis of visual acuity by irreversible neuronal damage [47]. But it remains possible that elevated CRP levels are more likely a surrogate marker for a more extensive initial microvascular insult with subsequently more severe macular edema rather than acting as an independent causal driver of macular edema or visual impairment. Although higher inflammatory marker levels were associated with worse baseline macular edema morphology, there was no difference in treatment burden, assessed by the required number of intravitreal anti-VEGF injections, indicative for preserved responsiveness to anti-VEGF therapy during systemic inflammation, arguing against a major role of systemic inflammation as a driver of treatment resistance and limiting the clinical utility of CRP to guide therapeutic decisions. But the use of different anti-VEGF agents in our study cohort, such as bevacicumab, ranibizumab, or aflibercept, might be a confounder of the number of intravitreal injections. Like CRP, PTX also belongs to the family of acute-phase proteins. But unlike CRP, PTX is mainly released by macrophages and vascular endothelial cells, thereby reflecting more directly the inflammatory state of the vasculature [48]. Other studies reported that in patients with RVO, serum levels of PTX3 were significantly increased compared to controls. There are only a few studies analyzing the role of CRP in RVO. A case series reported increased CRP levels in patients with RVO [49]. But there are no studies evaluating the impact of CRP on functional and morphological outcomes in RVO.

Notably, in patients with stenosis of the internal carotid artery, levels of CRP and homocysteine were highest in those patients with ocular ischemic syndrome. Furthermore, plasma levels of CRP and homocysteine were higher in patients with ocular ischemic syndrome compared to patients with RVO. These findings from other studies may indicate that CRP and homocysteine may be suitable indicators for retinal ischemia that is more pronounced in ocular ischemic syndrome than in RVO [50].

Herein, the conversion of RVO to an ischemic disease stage was assessed by the requirement of retinal laser photocoagulation after 12 and 24 months, as defined in the Materials and Methods section. We did not observe any impact of initial CRP levels on required laser treatment. But interestingly, we found a numerically slightly higher percentage of patients with hyperhomocysteinemia who received retinal laser photocoagulation within 12 months and even more within 24 months. Although a higher proportion of patients with elevated homocysteine levels required laser treatment compared to those with normal levels, this difference did not reach statistical significance. The statistical analysis of our data may be complicated by the relatively small sample size in the hyperhomocysteinemia group (*n* = 9) compared to those with normal homocysteine levels (*n* = 48), which limits the statistical power to detect a difference despite a clinically relevant effect. Therefore, these findings should be interpreted with caution and are considered exploratory and underpowered. But these observations might be relevant in guiding clinical interpretation and warrant further investigation in larger, prospective studies. Moreover, although laser photocoagulation in our cohort was guided by defined clinical (neovascularization) and fluorescein angiography criteria (extent of non-perfusion areas and/or presence of neovascularization), ischemic conversion was assessed using a treatment-based surrogate rather than a uniform angiographic classification, and findings related to ischemic conversion status and required laser treatment should therefore be considered exploratory. Also, other studies found that hyperhomocystenemia might be a potential indicator for ischemic central RVO in the absence of local or systemic disease in elderly adults [19]. A limitation of our study is that we only measured homocysteine and CRP levels in the acute stage of RVO and not in the further course of the disease or upon diagnosis of ischemic conversion. Since differentiation between ischemic and non-ischemic RVO in the acute disease stage is hampered by retinal hemorrhages that aggravate the interpretation of fluorescein angiography and the identification of non-perfused retinal areas by blocking the fluorescein signal, establishing prognostic values to identify patients who are at high risk for ischemic conversion, which can lead to severe complications such as vitreous hemorrhage or neovascular glaucoma, is highly relevant for clinical practice. Interestingly, it has been demonstrated that homocysteine levels in patients with RVO significantly increased in the convalescent period and reached significantly higher levels compared to controls. Six months after the occurrence of RVO, homocysteine levels were significantly higher in patients with ischemic central RVO compared to those with non-ischemic disease, underlining the potential of homocysteine levels to predict ischemic conversion in RVO [51]. Therefore, these studies and our results might help develop new strategies for clinical routines in detecting ischemic conversion earlier, aiming to start laser treatment earlier to prevent ischemic complications in patients with RVO. Determining homocysteine levels not only in the acute disease stage but also in later disease stages might further improve the detection of patients at a high risk of ischemic disease. Since there is evidence that hyperhomocysteinemia is associated with ischemic RVO, our findings about an association between homocysteine levels and the higher CRT of central RVO-related macular edema are completely novel. Homocysteine is considered a systemic parameter for vascular inflammation and endothelial damage. There is evidence from other clinical and experimental studies that high levels of homocysteine damage the endothelial cells of blood vessels and activate coagulation factors. By reducing relevant gasotransmitters, such as nitric oxide, and by overstimulation of NMDA receptors, inflammation and oxidative stress are promoted, both of which are relevant pathophysiologic steps in retinal vascular damage [52]. Additionally, it has been shown that homocysteine damages the retinal endothelial cell barrier with subsequent hyperpermeability, a key component in the pathogenesis of macular edema in RVO [53].

It is very important to notice that the increased systemic inflammation exhibited by elevated levels of CRP or homocysteine might be a result of sources other than RVO or retinal vascular damage, such as systemic inflammation as a consequence of rheumatologic diseases or local infections. But this subsequent generalized inflammatory state with elevated inflammatory markers and signaling molecules may be incorrectly directed to pre-damaged retinal vessels during RVO and thereby worsen capillary damage and ischemia [54]. This might, for example, explain why patients with rheumatologic diseases, such as systemic lupus erythematosus, which is accompanied by systemic inflammation and increased levels of CRP, are at high risk for retinal vascular occlusion [55]. The fact that the homocysteine levels are higher in patients with RVO compared with controls [56], but yet comparable to those observed in patients with other thrombotic or atherosclerotic vascular diseases highlights that increased levels of inflammatory parameters, such as homocysteine, reflect more a systemic pro-inflammatory, thrombotic, or atherosclerotic condition that affects various vascular beds, rather than being specifically related to RVO per se [57].

This study has some limitations. Given the retrospective observational design and the potential for unmeasured confounders, such as cardiovascular risk factors, systemic inflammatory diseases, and concomitant medication, as well as the etiological heterogeneity of RVO, the observed associations between systemic inflammation and macular edema should be interpreted as descriptive and hypothesis-generating rather than causal. Additionally, due to the relatively small sample size, particularly in the subgroup with hyperhomocysteinemia, and the absence of formal adjustments for multiple comparisons, our analyses have a higher risk of type I errors, and *p*-values close to 0.05 should be interpreted with caution. Furthermore, clinically relevant cut-off values of inflammatory parameters were used from some analyses, and the subsequent dichotomization of continuous inflammatory parameters might reduce statistical power and precision. As another consequence of the retrospective design, the timing of blood sampling, symptom onset, and duration of macular edema before treatment was not fully standardized, which might have influenced the levels of inflammatory markers and disease severity, contributing to temporal bias. Given the single-center tertiary-care setting and the absence of comprehensive demographic and socioeconomic data, external validity is limited, and the results cannot be generalized to other settings and populations.

Despite these limitations, this study provides exploratory real-world evidence, generating relevant hypotheses about the role of systemic inflammation in retinal vein occlusion. This highlights the need for future prospective multicenter investigations aiming to analyze the complex interplay between vascular pathology and systemic inflammation.

## 5. Conclusions

This study revealed that elevated indicators of systemic inflammation, such as CRP and homocysteine, were associated with the severity of macular edema secondary to RVO. Additionally, CRP levels correlated with a decline in visual acuity. Although these biomarkers did not influence the number of required intravitreal injections. The observed trend toward a higher need for laser treatment in patients with hyperhomocysteinemia was based on a very small subgroup and should be interpreted as exploratory. It might serve as a foundation for the hypothesis of a more ischemic phenotype in these patients that needs to be tested in future studies. These findings underscore the potential role of systemic inflammation in the pathophysiology of macular edema in RVO and the importance of the identification of potential prognostic markers for clinical outcomes in patients with RVO.

## Figures and Tables

**Table 1:** Descriptive data of patients with macular edema secondary to retinal vein occlusion.

Characteristics		
Sex (%)		
	Female	48% (33/68)
	Male	52% (35/68)
Age in years (mean ± SD)		64.5 ± 12.1
Laterality in %		
	Right	41% (28/68)
	Left	59% (40/68)
Type of retinal vein occlusion (RVO) in %		
	Central RVO	48% (33/68)
	Hemi-central RVO	15% (10/68)
	Branch RVO	37% (25/68)
Lens status in %		
	Phakia	79% (53/67)
	Pseudophakia	21% (14/67)
Glaucoma	in %	10% (7/67)
Arterial hypertension in %		53% (32/60)
Morphological parameters (mean ± SD)		
	Foveal retinal thickness (µm)	591 ± 277
	Average central retinal thickness (µm)	580 ± 227
	Average central retinal volume (mm^3^)	12 ± 3
Visual acuity in LogMAR (mean ± SD)		
	Baseline	0.73 ± 0.61
	After 12 months	0.46 ± 0.40
Number of intravitreal injections (mean ± SD)		
	After 12 months	7.5 ± 2.4
	After 24 months	13.7 ± 4.7
Inflammatory parameters (mean ± SD)		
	C-reactive protein in mg/L	3.70 ± 4.58
	Homocysteine in µmol/L	11.49 ± 4.70

## Data Availability

The data presented in this manuscript are not publicly accessible due to privacy restrictions.
